# Ensemble Learning, Deep Learning-Based and Molecular Descriptor-Based Quantitative Structure–Activity Relationships

**DOI:** 10.3390/molecules28052410

**Published:** 2023-03-06

**Authors:** Yasunari Matsuzaka, Yoshihiro Uesawa

**Affiliations:** 1 Department of Medical Molecular Informatics, Meiji Pharmaceutical University, Kiyose 204-8588, Japan; 2 Division of Molecular and Medical Genetics, Center for Gene and Cell Therapy, The Institute of Medical Science, The University of Tokyo, Minato-ku, Tokyo 108-8639, Japan

**Keywords:** DeepSNAP, ensemble learning, neural network, pharmacokinetics, regression model

## Abstract

A deep learning-based quantitative structure–activity relationship analysis, namely the molecular image-based DeepSNAP–deep learning method, can successfully and automatically capture the spatial and temporal features in an image generated from a three-dimensional (3D) structure of a chemical compound. It allows building high-performance prediction models without extracting and selecting features because of its powerful feature discrimination capability. Deep learning (DL) is based on a neural network with multiple intermediate layers that makes it possible to solve highly complex problems and improve the prediction accuracy by increasing the number of hidden layers. However, DL models are too complex when it comes to understanding the derivation of predictions. Instead, molecular descriptor-based machine learning has clear features owing to the selection and analysis of features. However, molecular descriptor-based machine learning has some limitations in terms of prediction performance, calculation cost, feature selection, etc., while the DeepSNAP–deep learning method outperforms molecular descriptor-based machine learning due to the utilization of 3D structure information and the advanced computer processing power of DL.

## 1. Introduction

Machine learning (ML) is a data analysis method that discovers associated regularities and rules through the repeated learning of past cases and data. ML methods can be classified into three types: supervised learning, unsupervised learning, and reinforcement learning [1]. Supervised learning methods involve making a computer learn the correct data and produce the correct output for the input data. In contrast, unsupervised learning methods do not set correct data, and hence the computer itself learns the data characteristics through a large amount of data. In reinforcement learning methods, a computer makes judgments so that the numerical value of the output result is the highest. Therefore, in ML, predictions and inference are made by applying the regularities obtained through learning to unknown and future cases. Furthermore, deep reinforcement learning combines deep learning (DL) and reinforcement learning and is used when a small amount of labeled data and a large amount of unlabeled data are prepared. Supervised learning is divided into two tasks: classification to sort learning data into predetermined classifications and regression to predict future values of continuous data [2,3]. The main purpose of classification is to predict the classification to which the data for analysis belong. However, the main purpose of regression is to make predictions based on trends in continuous values. Regression analysis is a statistical technique that examines the relationship between a result number and a factor number. At this time, the factor value is called the explanatory variable and the resulting value is called the objective variable. An ML model is a mechanism that outputs results for input data and analyzes the input data for evaluation and judgments based on some evaluation criteria. That is, ML is a system that realizes a mechanism equivalent to human learning with computers. Based on a certain calculation method or algorithm, in an ML method, a computer discovers patterns and rules from input data, and by applying those patterns and rules to data, it predicts data. DL is an ML method in which a neural network consisting of many layers is used. The concept of DL is focused on this multilayered neural network, but DL makes it possible for computers to extract feature values themselves when discovering patterns and rules, even if the feature amount is not set in advance. The pattern is recognized by checking how well the input pattern matches the prepared pattern. It is a big breakthrough that feature values are created by themselves without being instructed in this way. In DL in the field of image recognition, the main focus is the combination of a large number of images and a convolutional neural network (CNN), which is a mathematical model that mimics the neural circuits of the brain and returns an output for an input value. In a CNN, the neural network has convolutional layers and pooling layers that function by the characterization of two main factors: recognition in the “local receptive field” and “extraction with weight sharing” [4,5]. When humans recognize an object, they do not grasp the whole image at once but recognize the object little by little by extracting each partial area. The property that responds to the clipped area is called a local receptive field, and a characteristic of a CNN is that it responds to only a small part of the input data, such as the local receptive field. Weight sharing is a mechanism for recognizing the features deemed as important at a specific position in an image as having high importance at another position. CNNs have been applied for classification, object detection, and voice recognition in various fields, including quantitative structure–activity relationship (QSAR). In this review, we summarize the advances in and give an overview of novel QSAR systems, including CNNs, ensemble learning, supervised learning for regression, and molecular descriptor-based ML.

## 2. Classification of Images by a Neural Network

The term “neural” in neural networks stands for neurons, i.e., nerve cells. It is one of the ML methods that artificially reproduces the mechanism of the cranial nerves with a computer program. DL is a more complex version of these neural networks [6]. A neural network of artificial neurons can be regarded as a function that receives multiple data and outputs the calculated results. For example, if a photograph is captured with a 1-megapixel CCD camera, there are 1,000,000 pieces of data, and if colors are defined by red, green, and blue (RGB) values, the image comprises 3 million data points. By specifying the color information and positions of one-million-pixel data, the photograph can be reproduced. Inputting many data and creating a well-fitting function that can be judged well is supervised ML. By constructing a function that can be judged well, even if a new photo is input, it can be judged with a high probability. A structure called a multilayer perceptron is often used for a neural network, and a more complicated network with 10 or more intermediate layers is used in DL. The input layer has a large amount of data, and the data obtained by multiplying the numerical values by some coefficient and totaling them become the data of the middle layer in the first layer, and the second layer of the middle layer data is obtained by multiplying the data by some coefficient and totaling them. An output is derived from these data, but it involves a huge number of calculations. A function that can be judged by deriving the optimum value of the kind of coefficient combination that can be judged most correctly is completed. In addition, a recurrent network is a type of neural network that has a regressing, recurrent structure that can use the information previously incorporated into the model for predicting continuous data, such as time-series data and language data. DL is a neural network with a large number of intermediate layers. By increasing the number of hidden layers, highly complex problems can be solved and the prediction accuracy can be improved. However, DL models are sometimes called black-box models because they are too complex for humans to understand how the prediction is derived [7].

## 3. Evaluation of Predictive Models and Predictive Performance

In supervised learning, the collected data are divided into training data and evaluation data for learning. Thus, the separated data evaluation is called “cross validation” [8]. Supervised learning builds a model that makes predictions on other data after a machine learns from human labels for correct answers. The step of constructing a model with a “correct label” is performed using training data, and prediction is performed using evaluation data. When building a model using all training data, a model can be formed that can fit the data but never fit the unknown data that comes later—this phenomenon is called “overfitting” [9]. To prevent overfitting, the data at hand are divided into training data and evaluation data to build and predict the model. For example, in supervised learning for classification, when the evaluation data are classified by the prediction model built from the training data, the degree of correctness among the total data is evaluated. Thus, we consider a model, i.e., line of separation, that is too strict for some training data; however, the accuracy of prediction for different data decreases. Therefore, it is a good idea to balance the goodness of fit with the simplicity of the model. In statistical analysis, information content criteria are set to judge the balance. If the dimensionality or number of variables of the data used to build the model is very large, the combination of variables increases exponentially and the amount of calculations increases tremendously. Then, a sufficient learning performance may not be obtained with the data on hand. This problem is called the “curse of dimensionality”; that is, the ML efficiency decreases because of too many data dimensions [10]. To avoid this problem, multiple variables can be combined into one feature amount (feature amount creation) or the combination of effective feature amounts can be narrowed down (feature amount selection). Thus, overfitting occurs when the dimensionality of the polynomial is too large for the number of training data; that is, the coefficient *w* tends to take a large value. Therefore, if the coefficient can be restricted to a small value, overfitting can be suppressed. Regularization is a technique based on this idea [11]. A regularization term, namely a penalty term, is added to the sum-of-squares error given in Equation (1) that penalizes the coefficients for growing large (2). Here, λ is the regularization factor and it is an arbitrary value.

E(w) = 1/2 ‖Xw − t‖^2^ (1)

E(w) = 1/2 ‖Xw − t‖^2^ + λ/2‖w‖^2^ (2)

The larger the value of *w*, the larger is the error function. To find a *w* that minimizes Equation (2), we expand and differentiate with respect to *w* to obtain the following formula.

(3) $$W={\left(X^{T}X+\lambda I\right)}^{-1X^{T}t}$$

In Equation (2), λ/2‖w‖^2^ was added to the sum of squared errors, but the regularization factor can generally be expressed as shown in Equation (5):

(4) $$\frac{1}{2}\lambda \overset{M}{\underset{j=1}{\sum }}{\left(\left|wj\right|\right)}^{q}\leq \;\eta$$

Equation (2) corresponds to *q* = 2. Adding a regularization term means limiting the coefficient wj to the range of Equation (5).

(5) $$\overset{M}{\underset{j=1}{\sum }}{\left(\left|wj\right|\right)}^{q}\leq \;\eta$$

In supervised learning for regression, when performing regression of evaluation data with a model created with training data, the target of evaluation is the difference between the predicted value of the training data and the actual value of the evaluation data. Taking into account the evaluation value of the prediction result, such as the correct answer rate of the output classification and the regression value, we examine whether the model has actual versatility. Typical cross validation methods include the holdout method and the k-fold method [12,13]. The k-fold method is a cross validation method that divides the training data into several (*k*) pieces and repeats model construction and verification for the number of divided data (i.e., *k* times). One of the divided data groups is used for validation and the remaining data are used for model building. First, one data group is used for validation, and the first result is checked from the fit of the model built on the rest of the data. In the second round, another data group is used for validation, the rest of the data are used for model building, etc. Model construction and verification are performed for the number of divided data, and the average value of verification that is repeated *k* times is taken as the result. This prevents models from fitting only to specific data increases by testing different data multiple times for verification. The holdout method is a method adopted for confirming model accuracy and is performed by dividing one dataset into training data and evaluation data. Training data and evaluation data must be separated because they affect the accuracy of the model. In the holdout method, the data used as training data are never used as evaluation data. Similarly, the data used as evaluation data will not be used as training data. In this method, it is necessary to be careful not to confuse the data. Linear regression is a type of regression analysis that predicts the value of a target variable based on the values of another explanatory variable [14,15]. Predicting one target variable with one explanatory variable is called single regression analysis [16]. The relationship between the two datasets that make the prediction can be expressed in the form of a linear equation (Equation (6)), which is the most basic model used in regression [17,18]. If a (slope) and b (Y intercept) are known, *y* can be predicted from *x*. The accuracy of the prediction is expressed by the correlation coefficient, i.e., the coefficient of determination [19]. Analysis with two or more explanatory variables, i.e., two or more dimensions, is called multiple regression analysis [20,21]. Multiple selection of appropriate variables makes it possible to set up a prediction formula, i.e., Equation (7), that is easy to calculate and has few errors.

y = ax + b(6)

y = a1 × 1 + a2 × 2 + a3 × 3 + a4 × 4 ⋯⋯ + b0(7)

Furthermore, to improve the prediction performance of a model, it is necessary to minimize its generalization error, which can be divided into three parts, namely bias and variance, which are minimizable errors, and noise, which is an irreducible error. This division is called bias–variance decomposition [22,23]. Minimizing bias requires learning more from the training data. However, if the bias is too small, the variance will become large. In contrast, if the variance is reduced too much, the bias will increase. It is necessary to find an optimal solution that balances both. When minimizing two prediction errors, bias and variance, we simultaneously need to consider the tradeoff. Hence, this situation is called the bias–variance dilemma [24]. The relationship between bias and variance follows the relationship that if one side wins, the other side will not win, i.e., the bias–variance tradeoff [25]. Bias in ML and statistics model prediction refers to the difference between the predicted and true values, that is the bias error, arising from incorrect model assumptions, where bias is the error due to the simplification of the actual problem [26]. For example, linear regression simplifies the problem.

Variance refers to the spread of predicted values, that is the variance error, which arises from fluctuations in the training data [27]. If the model prediction has a lot of bias, the model cannot accurately represent the relationship between inputs and outputs. In other words, even training data cannot be predicted accurately; this phenomenon is called underfitting [28]. Additionally, if the variance in the model prediction is too large, the model has learned noise in the training data; in this case, unknown data such as test data cannot be accurately predicted and overfitting occurs [29]. For high accuracy, the variance must be kept low.

## 4. Ensemble Learning

Ensemble learning (EL) is a method of taking a majority vote and learning to improve the prediction ability for unlearned data by combining the data trained as individual learners [30,31]. It refers to training multiple models and outputting a predicted value by a majority vote or average. Two concepts, namely the bias and variance, are important in EL, which is learning to collect information with low accuracy and increase accuracy. However, if the accuracy does not improve enough, the balance between bias and variance may be poor. The bias is simply the difference between the actual and predictive values. The smaller the difference, the higher the accuracy and the more accurate is the prediction, i.e., low bias results in accurate values, resulting from inadequate training. Variance, on the other hand, simply means the degree to which the predicted values are scattered. A state in which the degree of dispersion is high is called a high variance state, and the accuracy is low in this case. The high variance is due to overfitting, which is caused by overtraining [32].

The model used for EL should be a weak learner, that is a learner with low accuracy when used alone, as the name suggests. In terms of the bias–variance tradeoff (there exists a tradeoff relationship between model complexity and simplification), although the bias–variance tradeoff will fall, if the model is too simple, the generalization performance of the training data cannot be improved, and often the bias is high and the variance is low. That is, a simple model that is not overfitted is obtained. In addition, the characteristics of EL are not only used simply for ML algorithms, such as regression and classification, but also as an auxiliary method when obtaining learning coefficients for other ML algorithms. The effectiveness of EL is that it can take a majority vote using weak learners. In the case of simple binary classification, when classification is performed with a normal classifier, if the classifier misclassifies, an incorrect result will be returned. However, since EL employs a majority decision, if there are m learners, the answer is corrected as long as (*m* + 1)/2 or more learners do not misjudge. For classification problems that are prone to mistakes, EL is very useful because it allows the results of multiple classifiers, such as neural networks, SVM, and naïve Bayes, to be true [33]. Furthermore, assuming that each weak learner is statistically independent, and assuming that the error judgment probability of each weak learner is uniformly *θ*, out of *m* weak learners, the probability of *k* false positives is as follows:

(8) $$P\left(k\right)=mCk\theta^{k}{\left(1-\theta \right)}^{m-k}$$

To simplify the explanation, consider that if *k* of *m* weak learners make mistakes, the smaller the value of *m*, the lower the mislearning rate [34]. One of the main EL methods, bagging is a method of training that uses some of the information in the training data rather than all of it and then combines all the training results [35]. Each training can be computed independently, thereby allowing parallel processing. Bagging involves selecting weak learners and merging them into the final learner using the bootstrap method. The basic bagging method is quite simple:

1. Repeat the following steps *B* times.
  - Create a new dataset by *m*-time split sampling from the training data.
  - Build a weak learner *h* based on the divided dataset.
2. Construct the final learning result using *B* times weak learners *h*.
  Classification: *H*(*x*) = arg max |{*i*|*hi* = *y*}| (9)
  (10) $$\mathrm{Regression}:H\left(x\right)=\frac{1}{2B}\sum_{i=1}^{B}hi$$

The formula for the part that finalizes the final learning result is given above in (10) and (11); in the case of classification problems, each weak learner is sorted so that the overall accuracy is the highest [36]. On the other hand, for regression, each weak learner is normalized by the overall value. An example of a well-known ML algorithm that uses bagging is the random forest algorithm. Using a part of the training data and merging it at the end is a common feature in bagging; boosting is the process of reusing previously used data to literally provide a boost. Hence, parallelism is not possible as with bagging. Boosting is an EL algorithm that sequentially builds weak learners.

In EL, high accuracy is also achieved by using many weak learners (e.g., decision trees) that do not have high accuracy alone. Bagging uses both the weak learners in parallel and the overall results of each model. In random forest, which combines bagging and decision trees, variance can possibly be suppressed by creating a number of decision tree models for the data and aggregating each result to output the final result.

## 5. DeepSNAP: DL and EL

**Figure 1:**
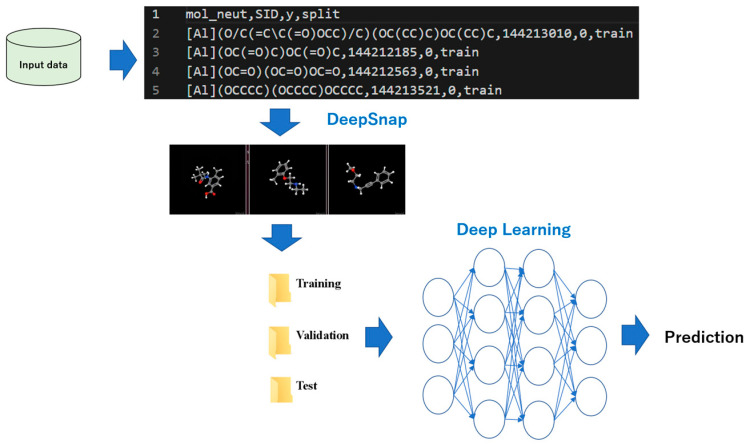
DeepSNAP–DL. The input data of a chemical compound are converted into the SMILES format, and snapshots are produced from different angles as image data. These data are split into three datasets, namely the training, validation, and test datasets, automatically. The prediction model is constructed by DL using these image data.

**Figure 2:**
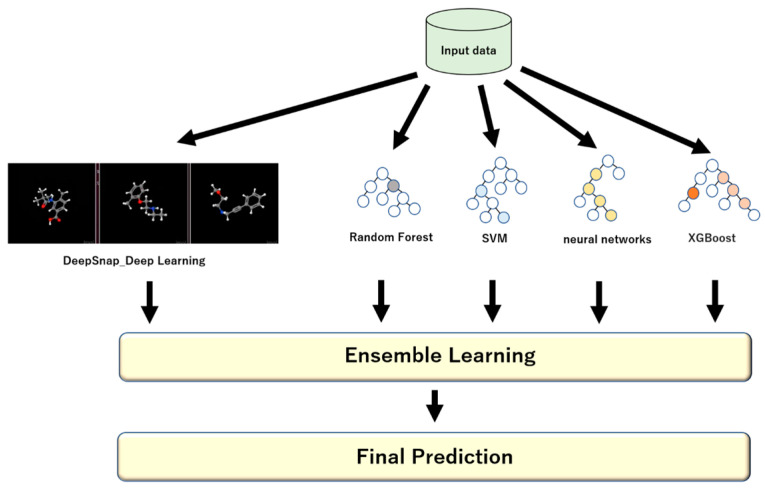
Ensemble learning with DeepSNAP–DL and descriptor-based ML, including random forest, SVM, neural networks, and XGBoost.

QSAR is a method for in silico prediction of chemical substances with physiological activity. In particular, it is one of the key components of integrated toxicology assessment systems, which are highly likely to cause adverse effects to chemical structures and are useful in prioritizing and narrowing down chemicals requiring safety assessment. It can also contribute to alternatives to or minimization of animal testing. In QSAR, the correlation between the structure and activity of compounds such as pharmaceuticals is determined quantitively as numerical values. These values are handled via supervised learning that calculates feature values using chemical information for compounds and builds prediction models [37]. Molecular descriptors, which are the characteristic quantities that reflect the structure of a compound in QSAR, include fingerprints that determine the presence or absence of partial structures and the measured and estimated values of the physicochemical properties of compounds [38]. Using the descriptors, QSAR models are constructed by several algorithms, including random forest, support vector machine, extreme Gradient Boosting (XGBoost), Bayesian networks, multiple linear regression, polynomial regression (PLR), decision tree regression, and neural networks [39,40]. However, modeling in QSAR has some limitations related to the prediction performance, feature selections, calculation cost, etc. Therefore, Prof. Uesawa developed a new deep learning-based QSAR system, DeepSNAP, which generates an omnidirectional snapshot depicting the three-dimensional (3D) structure of chemical compounds (Figure 1) [41]. In DeepSNAP, each chemical structure is optimized for steric conformation and portrayed to depict a ball-and-stick model with different colors representing different atoms. Using datasets of approximately 9000 chemical structures in the simplified molecular input line entry system (SMILES) format and the corresponding activity scores, which represent the agonist or antagonist levels of nuclear receptors and stress response proteins, from a database composed of high-throughput quantitative screening results, two datasets were prepared by defining “active” or “inactive” agonist or antagonist activities. The aforementioned database was derived from the Toxicology in the 21st Century (Tox21) 10k library composed of chemicals from commercial sources, such as pesticides, industrial chemicals, food additives, and drugs [42,43,44,45,46,47,48,49]. Then, the SMILES format was applied to a 3D conformational import to generate the SDF files of the chemical database. In this database, the 3D chemical structures of compounds were depicted as 3D ball-and-stick models and captured continuously and automatically as snapshots with user-defined angle increments on the x-, y-, and z-axes. This was done to extract the spatial and temporal features in the images. Finally, 256 × 256 pixel resolution PNG files (RGB) were saved and automatically split into three datasets, namely the training, validation, and test datasets. Prediction models were created by using training and validation datasets, and the performance with the test dataset was examined. In addition, by optimizing DeepSNAP parameters such as zoom factor, atom size, and molecular bond radius, the performance of the prediction models can be improved because of the powerful feature discrimination capability of DeepSNAP without the need for extracting and selecting features. Furthermore, a combined system of DeepSNAP–DL with molecular descriptor-based methods was reported to construct regression models of rat clearance (CL), i.e., in vivo pharmacokinetic parameters among the parameters of absorption, distribution, metabolism, and excretion. These models outperformed models based on molecular descriptor-based methods (Figure 2) [50,51]. DeepSNAP–DL predicted compound performance from correlations based on large experimental datasets of compounds. ML can perform a correct action in response to input data, collect feedback on how well it performed, and improve it. Despite the significance of CL prediction in the field of drug discovery, few in silico prediction systems with sufficient performance have been established to date. Therefore, this novel system using a combination of DeepSNAP and DL and the molecular descriptor-based methods will be a useful tool for CL prediction. In the ensemble method, a molecular descriptor is selected by DataRobot, in which the feature importance of the descriptors calculated by the permutation importance of the prediction models is more than 0.5 times the highest average effect [50]. Some available EL methods in QSAR that help improve the ML performance have also been reported [52,53].

## 6. Application of DL in New Drug Development and Medicine

By using EL consisting of DeepSNAP–DL with molecular descriptor-based methods, a prediction model system for CL, which can be regarded as the blood cleaning speed by the processed organ, with regard to pharmacokinetics (PK) parameters in absorption, distribution, metabolism, and excretion, indicated a high prediction performance [50]. Furthermore, a highly accurate regression analysis was achieved by incorporating DeepSNAP–DL probability as an explanatory variable into the descriptors of the conventional ML methods [51]. In building a CL prediction model using the DeepSNAP–DL method, conditions including four angles (65°, 85°, 105°, and 145°), five learning rates (0.0000001 to 0.001), and five maximum epochs (15 to 300) were considered. As a result, the receiver operating characteristic area under the curve (ROC AUC) with the highest prediction performance was calculated (0.8974) under the conditions of a learning rate of 0.000001, a maximum epoch of 300, and a 145° angle. In addition, the ROC AUC was calculated to be 0.943 in predictive model construction by the ensemble model method, in which the average value of the predicted probabilities obtained by the DeepSNAP–DL method and the descriptor-based random forest method was used as the predicted probability of a new combination of prediction models. Balanced accuracy (0.868), F-measure (0.845), and Matthew’s correlation coefficient (0.739) were used as the other evaluation indices. Prediction models are constructed by QSAR analysis for various prediction targets such as toxicity and pharmacokinetic parameters, but the problem is that the prediction accuracy is insufficient. This research focused on rat CL and developed a new prediction accuracy improvement method using the DeepSNAP–DL method with descriptors of conventional ML.

In addition, the application of artificial intelligence technology to the drug discovery field is expected to accelerate new drug development and realize innovative new drugs. However, because drugs and their target proteins have different types of structures, it is difficult to predict drug–protein combinations that are effective in treating diseases with high-throughput and high-performance. Therefore, based on the knowledge of chemistry and biology, molecular interaction prediction methods for drug development are being advanced by combining DL methods. In addition, because conventional methods that do not use the 3D structure of drugs or proteins cannot specify the interaction site from prediction results, there is a problem in interpreting the results. However, new technologies that can identify and visualize interaction sites are expected to accelerate the task of narrowing down new drugs from a large number of candidates. In addition, large-scale searches for drugs candidates by computers are expected to lead to the development of innovative new drugs that cannot be reached with human knowledge and experience alone. In general, drugs can be expressed as atoms and bonds as graph structure data, and proteins can be expressed as amino acid sequences as sequence structure data. Therefore, graph neural networks and CNNs, which are DL methods suitable for each drug data and protein data, respectively, are applied to each data point, by which feature vectors that appropriately capture the properties of the drug and protein are calculated. By learning this feature vector using large-scale data of drugs and proteins, it is possible to predict the presence or absence of interactions [54].

While DL can make fast and highly accurate predictions, it is difficult to interpret the prediction results. When dealing with chemical and biological data, it is necessary to judge the validity of results by comparing the results of automatic predictions made by computers with the chemical and biological knowledge already possessed by humans. Therefore, ML techniques with easy-to-interpret results are important.

## 7. Conclusions

A deep learning-based QSAR analysis, DeepSNAP, outperformed molecular descriptor-based conventional ML using 3D chemical structures and automatic extraction of features from image data. This system is mainly constructed of a neural network with multilayers, and it is characterized by a high performance of classification and object detection. Furthermore, by combining DeepSNAP–DL and conventional ML (such as EL), regression models can be constructed. The final prediction model obtained by combining multiple weak learning models is constructed. However, EL is not always a versatile learning method. It is important to consider that classifiers are diverse and accurate when bagging is used.
